# Chitosan Soft Matter Vesicles Loaded with Acetaminophen as Promising Systems for Modified Drug Release

**DOI:** 10.3390/molecules29010057

**Published:** 2023-12-21

**Authors:** Loredana Nicoleta Hilițanu, Liliana Mititelu-Tarțău, Eliza Grațiela Popa, Beatrice Rozalina Bucă, Irina Luciana Gurzu, Paula Alina Fotache, Ana-Maria Pelin, Daniela Angelica Pricop, Liliana Lăcrămioara Pavel

**Affiliations:** 1 Department of Pharmacology, Faculty of Medicine, ‘Grigore T. Popa’ University of Medicine and Pharmacy, 700115 Iasi, Romania; ln.rusu@yahoo.com (L.N.H.); beatrice-rozalina.buca@umfiasi.ro (B.R.B.); fotachepaula@yahoo.com (P.A.F.); 2 Department of Pharmaceutical Technology, Faculty of Pharmacy, ‘Grigore T. Popa’ University of Medicine and Pharmacy, 700115 Iasi, Romania; 3 Department of Preventive Medicine and Interdisciplinarity, Faculty of Medicine, ‘Grigore T. Popa’ University of Medicine and Pharmacy, 700115 Iasi, Romania; irina-luciana.gurzu@umfiasi.ro; 4 Department of Pharmaceutical Sciences, Faculty of Medicine and Pharmacy, ‘Dunarea de Jos’ University, 800010 Galati, Romania; anapelin@gmail.com; 5 Research Center with Integrated Techniques for Atmospheric Aerosol Investigation in Romania, RECENT AIR, Laboratory of Astronomy and Astrophysics, Astronomical Observatory, Physics, ‘Al. I. Cuza’ University, 700506 Iasi, Romania; daniela.a.pricop@gmail.com; 6 Department of Morphological and Functional Sciences, Faculty of Medicine and Pharmacy, ‘Dunarea de Jos’ University, 800010 Galati, Romania; doctorpavel2012@yahoo.com

**Keywords:** acetaminophen, lipid, vesicles, chitosan, biocompatibility, mice

## Abstract

Our study was designed to acquire, characterize and evaluate the biocompatibility of novel lipid vesicles loaded with acetaminophen (APAP) and coated with chitosan (CS). We investigated the in vitro and in vivo drug release kinetics from these systems, and we conducted assessments for both in vitro hemocompatibility and in vivo biocompatibility. For the in vivo biocompatibility evaluation, the mice were randomly divided into four groups of six animals and were treated orally as follows: control group: 0.1 mL/10 g body weight of double-distilled water; CS group: 0.1 mL/10 g body weight 1% CS solution; APAP group: 150 mg/kg body weight APAP; APAP-v group: 150 mg/kg body weight APAP-loaded lipid vesicles. The impact of APAP-v on various hematological, biochemical, and immune parameters in mice were assessed, and the harvested tissues were subjected to histopathological examination. The innovative formulations effectively encapsulating APAP within soft vesicles exhibited reasonable stability in solution and prolonged drug release in both in vitro and in vivo studies. The in vitro hemolysis test involving APAP-loaded vesicles revealed no signs of damage to red blood cells. The mice treated with APAP-v showed neither significant variances in hematological, biochemical, and immune parameters, nor structural changes in the examined organ samples, compared to the control group. APAP-v administration led to prolonged drug release. We can conclude that the APAP-v are innovative carrier systems for modifying drug release, making them promising candidates for biomedical applications.

## 1. Introduction

APAP is a derivative of para-aminophenol (N-(4-Hydroxyphenyl)-acetamide). It possesses analgesic and antipyretic properties, but does not exhibit significant anti-inflammatory effects. It acts by indirectly inhibiting the COX-1, COX-2, and COX-3, thus raising the pain threshold and reducing painful sensations [1,2,3].

After oral administration, APAP exhibits favorable absorption characteristics, with a peak concentration in the bloodstream achieved approximately 90 min following ingestion. Its maximum effect is typically observed between 30 min and 2 h after oral intake, while rectal administration results in a maximum effect within 3–4 h [4]. When taken orally, the duration of its action ranges from 3 to 7 h and results in a bioavailability of 88% [5,6]. The lack of total absorption resides in its property of low permeability, being a class 3 BCS drug (high solubility/low permeability). APAP binds to plasma proteins to a varying extent, typically around 10–25% [7,8]. APAP has a half-life ranging from 1 to 4 h, and its primary route of excretion is through the urine, with approximately 5% being eliminated in its free, unconjugated form [6].

In the existing literature, there are numerous considerations concerning the development of lipid vesicles to integrate drugs, constituting a broad area of research interest. Our methodology for crafting these systems stands out as an original approach that, as of now, has not been undertaken by any research team. In our chitosan-based lipid vesicles, APAP serves as the model drug, representing an innovative strategy. The uniqueness of chitosan-based vesicles encapsulating active substances stems from the combination of chitosan’s distinctive properties with the advantages inherent in vesicular drug-delivery systems [9,10]. A chitosan coating can improve the stability of lipid vesicles, protecting their cargo from degradation by enzymes or harsh environments [11]. This fusion presents opportunities for enhancing drug effectiveness, minimizing adverse effects, achieving targeted delivery, and improving patient adherence [12,13]. Researchers consistently delve into refining and optimizing these formulations to unlock their complete potential in pharmaceutical applications [14]. Hence, the studies highlighted in the paper align with the current landscape of modern research endeavors within this field.

The clinical use of APAP has demonstrated potential gastrointestinal irritation and related side effects. Encapsulating it within lipid vesicles offers a promising strategy to mitigate these adverse effects through controlled release and targeted delivery, potentially reducing direct interaction with the gastrointestinal tract. Tackling these challenges through the development of APAP-v represents a significant stride in pharmaceutical research.

Currently, there is a limited availability of modified-release acetaminophen tablets on the market, in particular, those containing 650 mg per tablet. Through the development of these vesicles, we are broadening the scope for novel extended-release acetaminophen formulations. These formulations may encompass a combined total of two therapeutic doses, aiming to address chronic moderate pain. They can be used either independently or in synergistic combinations with other analgesics.

Various methods and different active ingredients have been used to design nanosystems encapsulating acetaminophen. Using surfactant and cholesterol as ingredients, a group of researchers prepared multilamellar niosomes containing acetaminophen through the thin film hydration method [15]. Acetaminophen was also loaded into PEGylated nano graphene oxide particles via the sonication technique [16] or in core-shell biodegradable microspheres [17]. Other types of nanoparticles with acetaminophen based on poly(lactide-co-glycolide acid) were acquired using the emulsion-solvent evaporation procedure [18].

Since their discovery in the 1960s, liposomes have been studied intensively for the formulation of many dosage forms, in order to overcome some of the inconveniences posed by drugs for various administration routes. Liposomes [19] can trap hydrophobic and hydrophilic drugs, thus improving drug delivery and bioavailability, or modifying the drug-release kinetics.

In recent decades, a novel approach has surfaced involving the development of increasingly sophisticated liposomal vesicles within the realm of nanoformulation. These new drug-delivery systems are designed to target drug delivery to specific tissues and limit some of their side effects, especially liver toxicity [20,21].

Liposomes are lipid vesicles with a spherical morphology, containing a central hydrophilic core, surrounded by a phospholipidic membrane. The scientific community has shown increasing interest in liposomal systems, due to their biocompatibility, biodegradability, non-immunogenicity and lack of toxicity [22,23]; thus, multiple drug-delivery systems based on liposomes for various administration routes have been designed. However, liposomal systems have their limitations: rapid blood clearance when administered via intravenous injection, and the tendency to combine/fuse, causing drug leakage during storage [24,25,26]. One of the solutions to these problems is to induce surface changes (i.e., using a different agent to coat the liposomes), in order to enhance their stability, extend their life in the bloodstream and ensure modified release of the entrapped drug [27,28].

Many synthetic, semisynthetic or natural polymers (collagen, gelatin, quercetin) used for coating the liposomal surface have been investigated for their potential as drug carriers with possible use in the biomedical field. The main problem of the liposomal in vivo fate is their uptake by the reticuloendothelial system cells, which considerably lowers their bioavailability. The most implemented method to reduce this phenomenon is by coating the unilamellar lipid vesicles with hydrophilic substances (i.e., CS), which results in reduced phagocytic uptake, decreases the interaction with other proteins, and improves vesicle stability and transmucosal oral absorption [29]. It has been shown that the most effective process for the development of lipid-based drug carriers is sonication homogenization, which provides a narrow particle-size distribution, higher particle content in dispersions and avoids organic solvents. The systemic utilization of coating materials primarily offers crucial advantages such as maintaining physical stability, promoting dispersion, and ensuring the presence of colloidal particles in the bloodstream [30].

CS is a naturally occurring polysaccharide compound derived from marine crustaceans, mollusks, insects, and fungi; it has been studied intensively as a biomedical material [31,32,33], due to its anti-tumor, anti-ulcer, immune-stimulatory, antidiabetic, antioxidant, and antimicrobial properties [34]. It can be formulated in various dosage forms such as solutions, gels, mixtures, medicated sponges, tablets, membranes, pastes for different applications [35].

CS nanoparticles fulfill the requirements that are sought after in such systems, including effectiveness, affordability, compatibility with the body, degradability, lack of toxicity, and absence of immunogenic properties [35,36]. Also, the use of CS-coated nanoparticles has demonstrated some intrinsic properties, such as high antioxidant action, anti-hyperlipidemic and liver-protective action against fat consumption or alcohol-induced steatosis [37].

Swelling reduces liposomal stability and can cause the quick release of the enclosed agents (such as natural products, antibacterial substances, anti-inflammatory medicines, cardiovascular drugs, hormonal compounds); several studies have shown that the CS layer acts like a wall that prevents swelling, improving the stability of soft lipid vesicles [38]. As a linear polysaccharide with a positive charge, CS has the ability to create stable complexes with negatively charged compounds like phospholipids, making it a potential candidate for the entrapment and controlled delivery of active substances [33,39,40]. It is a cationic polyelectrolyte with a high proportion of primary amines, which can interact easily with various targeted surfaces, such as cell membranes or the lipid bilayer of vesicles [41]. CS is soluble in acidic aqueous solutions, in correlation with the pH value of the medium and the percentage of de-acetylation, due to the protonation of amino groups [42]. Differences in molecular mass, the degree of de-acetylation and in the structural position of residue acetyl groups on the main polymer chain can lead to different properties of CS grades [39,43].

The purpose of our study was the design, characterization and the biocompatibility evaluation of original CS-coated vesicles entrapping APAP.

## 2. Results and Discussion

From a physical perspective, lipid vesicles prove versatile in encapsulating both hydrophilic and lipophilic drugs, safeguarding the active substance from external factors. These concentric vesicles encase an aqueous compartment entirely enclosed by a lipid membrane, primarily composed of phosphatidylcholine and cholesterol, the latter serving as a fluidity buffer. However, one drawback observed in lipid vesicles against their usefulness is their susceptibility to instability in colloidal solutions, leading to agglomeration. To counteract this issue, we have successfully stabilized them through chitosan coating. Additionally, our findings indicate that these lipid vesicles containing a drug are leaky in nature, resulting in the rapid release of the active substance, emphasizing the necessity for the supplementary use of chitosan.

Coating lipid vesicles with hydrophilic substances like chitosan has resulted in decreased cellular uptake by the reticuloendothelial system and minimized interaction with other proteins. This coating of colloidal carriers has demonstrated enhancements in particle stability and the facilitation of oral transmucosal transport. It has been established that the most effective method for producing lipid-based drug carriers involves homogenization through sonication. This process ensures a precise particle-size distribution, increased particle content in dispersions, and eliminates the use of organic solvents.

### 2.1. pH Value of Solutions with APAP

It was noted that the addition of CS to the suspension with APAP-loaded vesicles led to a decrease in pH from 6.00 (for APAP solution) to 4.65 (for non-dialyzed APAP-v solution) (Table 1). Dialysis enabled both purification of the dispersion by removing the non-encapsulated drug residues and modification of the pH to values close to physiological values (pH of APAP-v solution = 6.63) (Table 1).

### 2.2. Size Distribution of APAP-v

**Figure 1:**
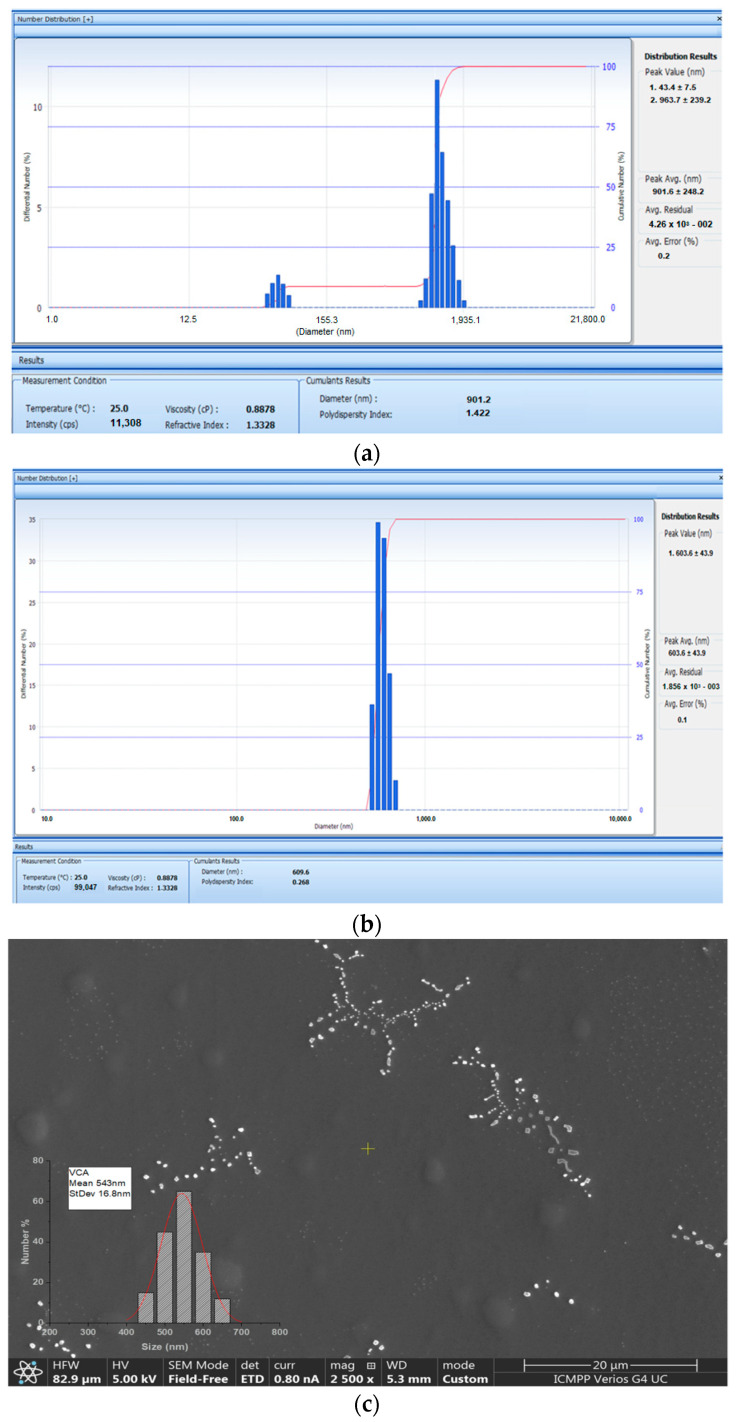
Size histogram (**a**) of APAP-v without CS and (**b**) APAP-v (**c**) SEM micrograph and size histogram of APAP-v obtained by direct measurement using Image J software, variant 1.8.0.

The image obtained with the Malvern Zetasizer Nano ZS ZEN-3500 device showed that for APAP-v without CS, the mean hydrodynamic distribution was situated at values of 901.2 nm (Figure 1a) and for APAP-v, the mean hydrodynamic distribution was at 603 nm (Figure 1b). The polydispersity index of 0.268 indicated a high degree of monodispersity, suggesting the presence of vesicles with fairly close dimensions, as seen in the size histogram (Figure 1a).

SEM micrographs of dialyzed APAP-v suspensions showed clusters of vesicles with relative spherical shapes and sizes ranging between 500 and 900 nm (Figure 1).

The SEM image highlights the arrangement of vesicles in clusters with branching structures of different lengths. The size histogram obtained from SEM micrograph analysis suggests the formation of vesicles larger than 543 nm (Figure 1b). It can be seen that the vesicles containing the drug were roughly round in shape and well dispersed. On the other hand, the presence of blisters that did not contain medicine, with irregular and elongated shapes could also be observed. The hydrodynamic size revealed by the diameter distribution led us to conclude that there was only one type of vesicles, with an average size of 609 nm.

### 2.3. Zeta Potential of APAP-v

The stability of colloidal solutions is defined according to the average zeta potential value. Zeta potential determination is a significant technique for the characterization of nanocrystals to estimate surface charge, which can be used to understand the physical stability of nanodispersions [44].

The stability of the APAP-v was assessed using a zeta potential stability model designed for positively charged spherical particles suspended within a uniform medium. According to this model, the affinity between particle surface and water generates a zone that is organized in layers of water, and this new structure highly modifies the particle–water–polymer properties. The electrostatic field on the particle surface, in combination with the thermal motion of the surrounding ions, creates opposite charges and therefore shields the electric charge on the particle surface. The actual electric charge of the diffuse shielding layer is equal in magnitude to the actual surface charge, but has the opposite charge [45].

**Figure 2:**
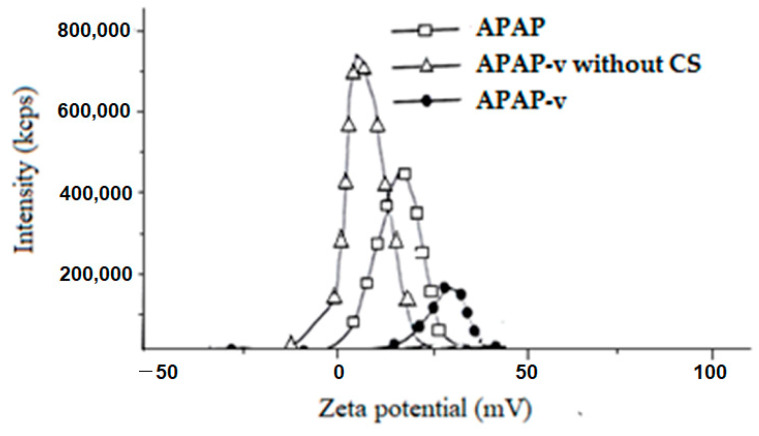
Zeta potential of APAP-v.

Based on the zeta potential distribution, our conclusion is that all the vesicle systems exhibited a predominant positive charge. In the case of the APAP solution, the measured zeta potential was +18.26 ± 1.43 mV, thus corresponding to an average level of dispersion stability. The average zeta potential value of +5.61 ± 0.36 mV of APAP-v without CS indicating that the colloidal solution had a low dispersion, being at the threshold of agglomeration (Figure 2). It was observed that, by coating the lipid vesicles with CS, the zeta potential was +28.45 ± 2.75 mV (Figure 2), suggesting a moderate stability level of the colloidal dispersion. We believe that coating the APAP vesicles with CS results in an augmentation of the positive charge on the vesicle surfaces and an escalation of the repulsive forces acting between them.

The physicochemical analysis of the obtained systems revealed that coating the APAP-loaded vesicles with a 1% CS layer enabled large vesicles of over 500 nm with very low polydispersity to be obtained, thus being quite uniform and having a spherical morphology. Following the stabilization of the lipid vesicles with CS, the zeta potential of 28.45 mV indicated a medium level of stability in the dispersion. The significant vesicle size could potentially result in reduced drug release into the tissue but the zeta potential can counterbalance this effect, ensuring effective permeability of the liposomes through the mucosal tissue. By using a neutral lipid, such as phosphatidylcholine, to obtain the vesicles, further coating them with CS led to an increase in the positive charge on the vesicle surface. Increasing the zeta potential led to higher electrostatic repulsion forces between APAP vesicles, with maintenance of dispersion stability.

The dispersions remained transparent for over six months after being prepared and stored at room temperature. This prolonged clarity might be attributable to the effective coating with 1% CS, which appears to safeguard the phospholipid membranes against oxidation when stored under various temperature conditions.

It was apparent that the new APAP-v demonstrated enhanced properties compared to APAP-v without CS, despite similar drug encapsulation efficiency. These improvements were characterized by notably smaller sizes (603.6 ± 43.9 versus 901.6 ± 248.2), a more uniform size distribution (polydispersity index 0.268 versus 1.422), and enhanced stability in the colloidal solution (zeta potential +28.45 ± 2.75 mV versus +5.61 ± 0.36 mV). An elevated polydispersity index of 1.422 suggested an inhomogeneous morphology of the APAP-v without CS. The efficiency of the release of the drug in the tissues is conditioned by a polydispersity index as low as possible, so as to avoid the agglomeration of the vesicles. The reduced size of APAP-v and its remarkably low polydispersity index supported their outstanding dispersion within the biological release medium.

### 2.4. The Efficacy of APAP Entrapment in Vesicles

**Figure 3:**
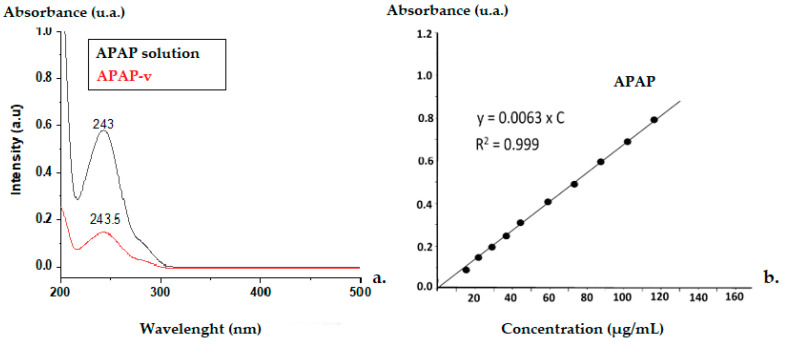
(**a**) UV spectra for APAP-v and APAP-solution (a.u.—absorption units) (**b**) calibration curve.

The calibration curve obtained of APAP in aqueous solution had the mean square deviation (R^2^) value of 0.999 (Figure 3). The UV spectra revealed that the APAP solution had maximum absorbance at 243 nm, while the APAP-v exhibited a maximum absorbance at 243.5 nm. Since the initial mass of APAP in solution was 16.67 mg/mL, and 3.48 g/mL of APAP was removed from the APAP-v, we assessed a 79.12% efficacy of drug entrapment in these microsystems.

### 2.5. In Vitro Release Kinetics of APAP from APAP-v

Studying the release kinetics in vitro, we found a slower release of APAP from the nanovesicles stabilized with CS, in comparison to the drug release pattern from the solution.

**Figure 4:**
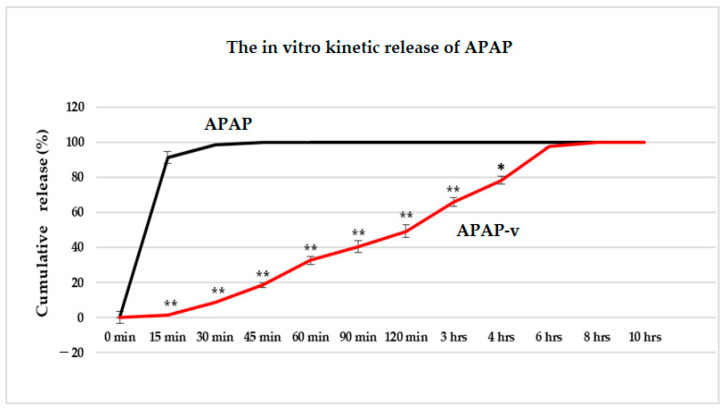
The in vitro kinetics profile (cumulative release percentage versus time) of APAP from APAP solution and from APAP-v, obtained using the permeation method. * *p* < 0.05, ** *p* < 0.01.

In the dissolution testing, we noticed that 91.4% of APAP was released from the solution after 15 min, while only 1.6% was released from APAP-v (Figure 4). At 30 min, it was revealed that from the simple solution, the drug was released in a proportion of 98.5%, and after 45 min the drug was completely released (Figure 4). This is in accordance with the standards set in the compendial guidelines for the release of APAP from solid dosage forms (e.g., tablets). Almost half of the amount of APAP (49.3%) was released after two hours from the APAP-v, 97.4% after 6 h, and the total amount after 8 h (Figure 4).

The finding that APAP was released in a slightly lower percentage from the vesicles may be ascribed to the enhanced dispersion of the drug as individual molecules tightly enclosed within the nanovesicles stabilized with CS. In our experimental setup, laboratory animals were not exposed to isolated lipid vesicles but rather to a colloidal solution in which these nanovesicles were dispersed.

### 2.6. In Vivo Release Kinetics of APAP from APAP-v

**Figure 5:**
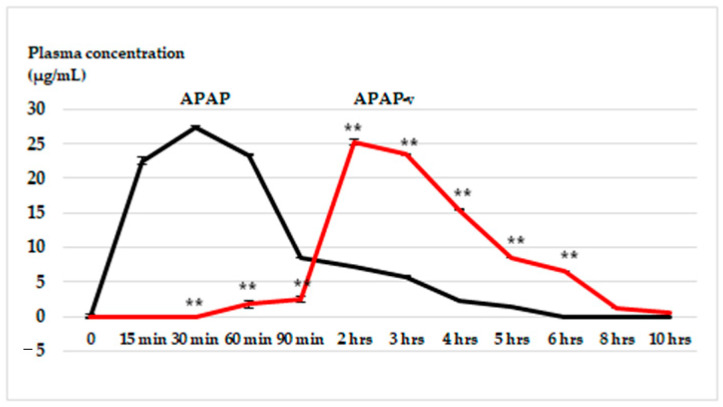
The in vivo kinetics profile (plasma concentration versus time) of APAP from APAP solution and from APAP-v, obtained using the HPLC method. The data are displayed as arithmetic mean ± S.D. of the average values for 6 animals per group. ** *p* < 0.01.

The highest plasma concentration of APAP following oral administration of APAP-v occurred at the two-hour mark (25.3 ± 1.17 µg/mL), in contrast to APAP-treated animals, where the maximum blood concentration (27.4 ± 1.21 µg/mL) was achieved at 30 min (Figure 5). Peak APAP levels were observed within the 15–60 min time frame, while notably elevated plasma-APAP concentrations resulting from drug release from APAP-v were seen between the two- and four-hour mark (Figure 5). This observation highlights a sustained release of the drug from the microsystems.

In the in vitro dissolution tests for both APAP and APAP-v, the outcomes are presented as the percentage concentration over a specified duration. Conversely, the in vivo release graph depicts results in plasma concentrations measured in µg/mL. To make comparisons uniform, converting these values to a percentage of the drug released allows for a direct assessment. In the in vivo assessment, at 1.5 h, the release of APAP from the vesicles measures 2.5 µg/mL in plasma, approximately equivalent to an 8% release of the drug from the vesicles. This rate of release appears significantly slower compared to the in vitro test, which demonstrates an approximate 40% release at 1.5 h.

In comparison to the in vitro release pattern of the vesicles, the in vivo kinetics exhibited a significant release after 2 h, reaching nearly 80%. This pattern resembles a gastro-resistant release model, albeit incomplete. The release of some APAP prior to 2 h can be attributed to the free fraction of APAP surrounding the vesicles. Observations at the 6 h mark on the in vivo graph indicated that approximately 20% of the drug remained, indicating a slow clearance of APAP from the body, with complete clearance occurring after 10 h. This extended duration contrasts with the in vitro test, indicating a prolonged presence of APAP in the system. Overall, even if the in vitro/in vivo graphs do not overlap, there is clearly an in vivo prolonged release of APAP-v.

A recent study revealed that utilizing a high molecular weight chitosan with elevated viscosity and a high deacetylation index, as employed in our lipid-vesicle production, leads to reduced solubility in the acidic conditions of the stomach [46]. Previous research demonstrated that a higher deacetylation index in chitosan results in increased steric hindrance, promoting the formation of a robust electrostatic complex with bile salts, thereby diminishing their efficacy [47]. The notable contrast between the brief drug-release duration of APAP and the extended latency period of APAP-v may be elucidated by the swift disintegration of lipid membranes in the gastric setting in comparison to the resistance created by chitosan with a high deacetylation index.

Conventional lipid vesicles are sensitive to damage caused by harsh chemical and enzymatic gastro-intestinal environments, resulting in reduced oral bioavailability [48]. The chitosan layer improved liposome stability in simulated gastric fluid [49], explained by the enhanced interactions between the chitosan and liposome surface under low pH in simulated gastric fluid (pH 1.2) due to the amino groups protonation in chitosan (pKa 6.5). The molecular configuration of chitosan also became more expanded, leading to a stronger affinity for the liposome surface [39]. The sustained release of encapsulated drug results from drug diffusion from the lipid bilayer and the adhesive chitosan layer [50]. Liposomes coated with chitosan released the drug in a slower manner, with this effect being attributed to the existence of the chitosan layer, which delayed the drug diffusion into the medium.

### 2.7. In Vitro Hemocompatibility

When the erythrocyte suspension was exposed to Triton X-100, a significant breakdown of the cell membrane occurred, leading to a notable level of hemolysis (88.45 ± 4.17%) (Table 2).

This difference was statistically significant (** *p*  <  0.01) when compared to the negative control group (0.07 ± 0.01%). Following the incubation of the erythrocyte suspension with the CS solution, only a slight degree of hemolysis was observed (1.64 ± 0.15%) (Table 2). This reduction was not substantially different from the saline solution group, indicating favorable in vitro hemocompatibility. Upon exposure to APAP, the red blood cells exhibited minor hemolysis (2.38 ± 0.25%), nonsignificant compared to the negative control group, indicating no harmful effect of APAP on the erythrocytes (Table 2). Incubating the erythrocyte suspension with APAP-v resulted in negligible hemolysis (2.52 ± 0.21%), without notable differences from the saline group (Table 2). This finding indicates that APAP entrapped in APAP-v did not induce the destruction of these blood elements.

Considering the literature-reported data, a hemolysis rate below 5% is regarded as acceptable, while values below 2% are deemed biologically insignificant. This implies that the administered substance can be safely used in vivo [51,52]. Scientific investigations have indicated that using CS with a high degree of deacetylation (exceeding 80%) is associated with a reduced risk of erythrocyte hemolysis [53]. Furthermore, when CS possesses a high molecular mass, its chains have low mobility, which greatly reduces binding to red blood cells [54,55]. Since CS is administered in a colloidal solution form, its impact on red blood cells is minimal. The finding that APAP-v exhibited low hemolysis suggests that these nanovesicles are compatible with blood and do not present a systemic toxicity concern.

### 2.8. In Vivo Biocompatibility

Following the preparation of APAP-v, we assessed their biocompatibility by examining specific serum parameters. In vivo studies serve as initial investigations that can illustrate the impact of administering these novel systems on plasma parameters. These indicators are pertinent for estimating the potential presence of liver inflammation or kidney dysfunction, oxidative processes, and any eventual structural changes in organs.

#### 2.8.1. Hematological Tests

**Figure 6:**
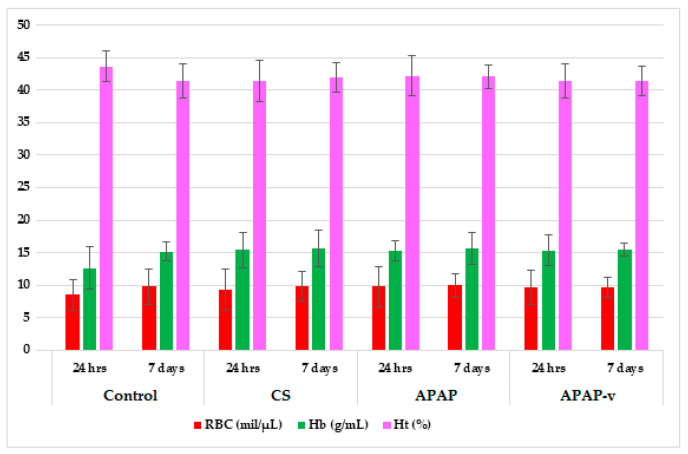
Hematological parameters: red blood cells (RBC), the hemoglobin (Hb) and hematocrit (Ht) values in CS, APAP, APAP-v groups. The data are displayed as arithmetic mean ± S.D. of the average values for 6 animals per group.

No statistically significant variation in RBC counts were detected among the groups of mice receiving CS, APAP, or APAP-v, when compared to the reference batch with distilled water at 24 h and at one week during the laboratory tests (Figure 6). Similarly, no momentous fluctuations in blood Hb were found among the batches studied in comparison to those receiving the control substance during the evaluation (Figure 6). Additionally, no noteworthy changes in Ht values were observed between the groups with entrapped and non-entrapped APAP; respectively, the group with polymer solution and the control lot, at the two time points designated for measurement (Figure 6).

The use of the polymer solution did not cause any statistically relevant deviation of the differential leukocyte count compared to the animals that received distilled water (Table 3). Likewise, there were no major variations observed in the white blood cells between blood samples from mice treated with APAP and APAP-v and those from the control batch (Table 3).

Determining the activity of certain liver enzymes helps to diagnose and monitor liver disease or damage. The tests measure the levels of certain enzymes and proteins in the blood. These liver function tests quantify the activity of specific enzymes that liver cells release in response to injury or disease [56].

**Figure 7:**
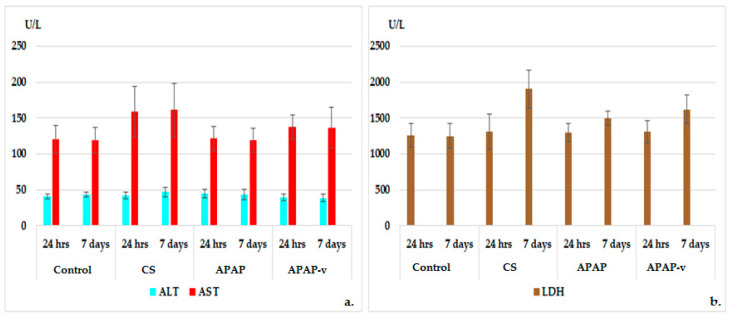
Liver enzymes: ALT, AST (**a**) and LDH (**b**) profile in CS, APAP, APAP-v groups. The data are displayed as arithmetic mean ± S.D. of the average values for 6 animals per group.

The administration of CS and APAP did not exhibit any statistically significant alterations in ALT and AST values when compared to the group receiving distilled water after 7 days (Figure 7). Furthermore, the hematological assessments did not show noteworthy deviations in ALT and AST values in the groups that were administered APAP-v versus the control group at any of the time points during the evaluations (Figure 7).

These observations indicate that the use of these substances does not impact liver function.

**Figure 8:**
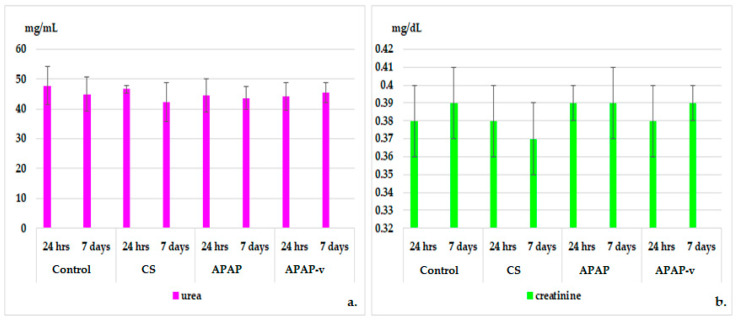
Serum urea (**a**) and creatinine (**b**) profiles in CS, APAP, APAP-v groups. The data are displayed as arithmetic mean ± S.D. of the average values for 6 animals per group.

The measurement of blood creatinine and urea levels was performed to assess kidney function because their values reflect the glomerular filtration rate; a parameter that defines kidney function. Regardless of its cause, kidney disease is associated with decreased glomerular filtration rate, and the severity of kidney disease correlates closely but indirectly with glomerular filtration rate [57]. There were no significant alterations observed in the plasma urea and creatinine profile in the animals treated with polymer and APAP in comparison to those receiving distilled water, either at 24 h or 7 days, in the experiment (Figure 8). Similarly, the use of APAP-v did not exhibit any apparent changes in blood urea and creatinine values when compared to the control group at these two designated time points (Figure 8). These findings are consistent with the absence of any renal toxicity.

**Figure 9:**
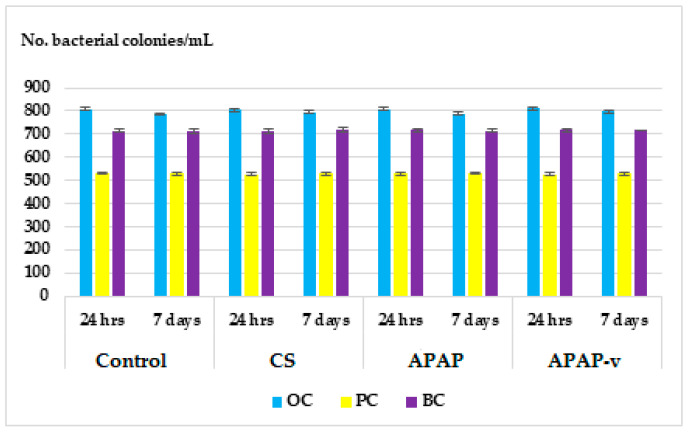
Effects of CS, APAP, APAP-v on OC, PC and BC levels in mice. The data are displayed as arithmetic mean ± S.D. of the average values for 6 animals per group.

The acquired immune response is mediated by antigen-specific cells and molecules that often interfere with components of the innate defense system [58]. During opsonization, antibodies bind to bacterial surface antigens to facilitate their phagocytosis by macrophages, which in turn can process and present protein antigens to specific T cells during a primary immune response. Antigen-specific lymphocyte responses are characterized by their proliferation and subsequent differentiation of function, such as the production of soluble mediators including antibodies and cytokines and the development of antigen-specific memory [59]. There were no significant changes in OC, PC and BC in mice receiving CS and APAP compared to animals in the distilled water group, either at 24 h or at 7 days after administration (Figure 9). No significant changes in OC, PC and BC in mice treated with APAP-v compared to distilled water group were detected after 1 day or 7 days (Figure 9). These results suggest that the substances do not exert any discernible effects on the animals’ immune systems.

#### 2.8.2. Histopathological Examination

**Figure 10:**
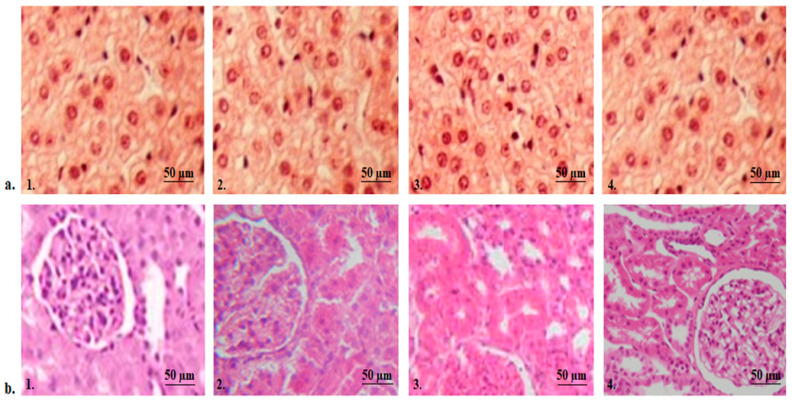
Histopathological images of liver (**a**) and kidney (**b**) tissue in the control (**a1**,**b1**), CS (**a2**,**b2**), APAP (**a3**,**b3**) and APAP-v (**a4**,**b4**) groups. H&E stain × 20.

The histopathological examination of liver tissues from mice receiving the test substances is an additional method of assessing the toxic potential of the administered compounds, taking into account that these systems are original, being obtained using an innovative nanoformulation technique. The analysis of liver fragments from the control animals showed hepatocytes without structural changes, the central vein and hepatic sinusoids having a normal configuration (Figure 10(a1)). Histopathological analysis revealed minimal alterations in the liver structure of animals subjected to CS (Figure 10(a2)) and APAP treatment (Figure 10(a3)) compared to the group of animals that received distilled water (Figure 10(a1)). Light microscopic examination of the liver fragments did not reveal any considerable disturbance of the liver architecture in animals treated with APAP-v (Figure 10(a4)) compared to the group receiving distilled water (Figure 10(a1)). No inflammatory infiltration or fibrosis was observed in any of the tested groups.

Histopathological investigation of kidney fragments from mice treated with double-distilled water revealed the normal renal architecture of renal corpuscles, no shrinkage or fragmentation of glomeruli and no degeneration or necrosis of renal tubules (Figure 10(b1)). There were no apparent changes in the kidney structure observed in animals treated with CS (Figure 10(b2)) and APAP (Figure 10(b3)) in comparison to the control group (Figure 10(b1)). Likewise, the use of APAP-v (Figure 10(b4)) did not lead to any significant disruptions in kidney morphology when compared to mice in the group receiving distilled water (Figure 10(b1)).

These results support the biochemical data we collected, which were utilized to indirectly confirm the intact functional state of the liver and kidneys.

## 3. Materials and Methods

### 3.1. Animals

The research was carried out in the CEMEX laboratories of the University of Medicine and Pharmacy “Grigore T. Popa” on animals obtained from the “Cantacuzino” National Military Medical Institute for Research and Development, Baneasa Station, Bucharest, Romania. Twenty-four healthy, genetically unaltered, pathogen-free white Swiss mice (aged 6–8 weeks) of both sexes, weighing 25–30 g, were chosen randomly to be used in the experiment.

The animals were housed in individual cages, at a controlled temperature (21 °C ± 2 °C), with relative humidity of 60–70% and alternating day/night regimen, with water and food available ad libitum, excepting the experiment period. The tests were performed throughout during the light period, in the time interval of 8–12 a.m. daily, to exclude chrono-biological interferences.

The mice were sacrificed under general anesthesia with 3% isoflurane at the end of the experiment, in accordance with the standard rules for the euthanasia of laboratory animals. The research protocol was approved by the Ethics Commission of the “Grigore T. Popa” University of Medicine and Pharmacy of Iasi (Certificate No. 24/14.07.2020), and performed in compliance with the national [60] and international [61] guidelines for handling laboratory animals.

### 3.2. Substances

APAP (Mn = 151.16, catalog code 1003009), the lipid L-alpha phosphatidylcholine Egg Yolk specific type XVIE (99% purity TLC, catalog code P 3556), CS (from crab shells, catalog code 448877), chloroform (Mn = 119.38, catalog code 288306), acetic acid (Mn = 60.05, catalog code A6283) were obtained from Sigma-Aldrich Chemical Co, Steinheim, Germany. The characteristics of CS used were: N-acetylation degree 82%, average molecular mass Mn = 94.810, average gravimetric mass Mw = 309.900 and the polydispersity index 3.26. The CS solution was prepared in 10% (*w*/*w*) acetic acid. The Model UltraMatic PLUS DI apparatus (Wasserlab, Navarra, Spain) was used to prepare double-distilled water.

### 3.3. The Design of APAP-v

Lipid vesicles exhibit considerable diversity in their shape, surface electrical charge and lipid composition, factors that influence variances in permeability across biological membranes [62,63,64]. The configuration of the lipid bilayer has a pivotal role in regulating the fluidity of the vesicle membrane. Also, it contributes significantly to both the stability of the vesicles and the pattern of drug-release kinetics when encapsulated [65,66,67].

An original method was employed to create APAP-v, which were further stabilized with chitosan (CS) for the purpose of modified drug release in laboratory animals [68]. The process involved the following steps:

- L-alpha-phosphatidylcholine (0.0075 g) was dissolved in 1 mL chloroform, and the solvent was evaporated using a Rotary evaporator RE-2000A (Ya Rong Biochemical Instrument Factory—Shanghai, China). This resulted in the formation of a dry lipid film.
- APAP (250 mg) was dissolved in 1 mL ethyl alcohol, and then diluted with double-distilled water up to 10 mL.
- The dry lipid film was rehydrated using the hydro-alcoholic APAP solution.
- The mixture was subjected to an ultrasonic field (25% amplitude) for 20 min at 29 °C, corresponding to an energy input of 20,000 kJ, using Bandelin 2450 SONOPULS ultrasonic homogenizers (Sigma-Aldrich- Steinheim, Germany). The lipid vesicles entrapping APAP were obtained.
- To coat the lipid vesicles with CS, 4 mL of a 1% CS solution was added to the dispersion of APAP-loaded vesicles. The mixture was magnetically stirred at 800 rpm for 10 min.
- The addition of acetic acid solution to the CS vesicle dispersion led to an acidic pH, enabling the protonation of the amino groups in the CS chain and facilitating its dissolution in water.
- The resulting dispersion was dialyzed for 2 h in double-distilled water, using tubular fiber membranes (type D6191-25EA) with a pore size of 12,000 Da MWCO (Sigma-Aldrich Chemical Co, Steinheim, Germany). This step aimed to achieve a pH value as close as possible to physiological levels, and the pH was monitored using a Sartorius Professional PP-50 pH meter from Sartorius Lab Instruments GmbH & Co. KG, Göttingen, Germany.

### 3.4. Characterization of Vesicles Entrapping APAP

The characterization of the obtained dispersions consisted of the analysis of structural and stability properties. Measurements of size distribution and stability of APAP-loaded vesicles was performed using a Malvern Zetasizer Nano ZS ZEN-3500 Apparatus (Worcestershire, UK), to assess their size and zeta potential.

For measurement of the electrophoretic mobility, the samples underwent dilution with 0.1 mM NaCl solution and were subsequently introduced into the measuring cell. The zeta potential was determined using the Smoluchowski equation. Each sample was analyzed in triplicate.

For the in vitro dissolution testing and obtaining the calibration curve, APAP was dissolved in ethyl alcohol to a concentrated solution (stock solution), which was further used to prepare six different dilutions. The UV–VIS spectra of the samples were measured using a Hewlett Packard 8453 UV-VIS spectrophotometer (Waldbronn, Germany). The absorbance was determined at 243 nm and the concentration was established according to the calibration curve of APAP. These spectra were recorded to highlight the inclusion of substances within vesicles and to obtain the release kinetics. The 2 h dialysis process was carried out to remove the organic solvents from the vesicles entrapping APAP (APAP-v). Following centrifugation, the precipitate containing APAP-v was dissolved in ethanol, effectively removing APAP from the vesicles. To determine the electrophoretic mobility, the samples underwent dilution using a 0.1 mM sodium chloride (NaCl) solution and were subsequently introduced into the measurement cell. Each sample was assessed three times to ensure accuracy. After the vesicles were degraded with chloroform, the solutions were centrifuged.

The calculation for determining the concentration of APAP loaded into the vesicles can be expressed as follows: The efficiency of APAP encapsulation (Ee) can be calculated as a percentage using the formula [69]:

Ee (%) = [(Wi − We)/Wi)] × 100.

where

Wi—the initial mass of APAP,

We—the mass of APAP released from the vesicles.

Scanning electron microscopy (SEM) was used to capture micrographs of APAP-v. The instrument used for this purpose was the SEM EDAX-Quanta 200 (Eindhoven, Germany). Measurement of the average size of more than 200 vesicles was performed by SEM micrographs using ImageJ analysis software, variant 1.8.0.

### 3.5. In Vivo Release Kinetics of APAP from APAP-v

To create an in vivo kinetic profile, two sets of six animals were administered a single oral dose using an esophagogastric device in the following manner:

Group 1 (APAP): 150 mg/kg body weight APAP;

Group 2 (APAP-v): APAP, 150 mg/kg body weight entrapped in lipid vesicles stabilized with CS;

To evaluate the in vivo kinetics profile, sequential 0.3 mL blood samples were obtained from mice under 1% isoflurane anesthesia. These samples were collected at various time points: initially at the starting point (moment zero) and subsequently at 15 min, 30 min, 60 min, 90 min, 2 h, 3 h, 4 h, 5 h, 6 h, 8 h, and 10 h after the oral administration of both entrapped and non-entrapped APAP. The concentration of APAP released into the bloodstream was determined using the high-performance liquid chromatography method (HPLC) conducted with an Agilent 1100 HPLC system (Agilent, Santa Clara, CA, USA). The UV detector of the HPLC was set to the same 243 nm wavelength as used in the in vitro assessment of APAP release.

### 3.6. Assessing the In Vitro Hemocompatibility of Vesicles Entrapping APAP

For the standard hemolysis assessment, a volume of 0.2 mL of freshly obtained blood from the lateral tail vein was collected into vacutainers containing heparin. The blood sample was then combined with sterile physiological serum at a ratio of 4:5. As part of the experimental setup, sterile physiological serum served as a negative control, and Triton X-100 (catalogue code X-100, Sigma-Aldrich Chemical Co, Steinheim, Germany) at 10% (*v*/*v*), an agent known for its hemolytic activity, was used as a positive control [70]. The mixtures were put to incubate for 45 min at 37 °C. Following this incubation period, 0.2 mL of extraction medium from each sample was placed in a water bath at 37 °C for one hour. Subsequently, each tube underwent centrifugation for 10 min at 1000 RCF (relative centrifugal force). The absorbance of the resulting supernatant was measured at a wavelength of 545 nm using a Hewlett Packard 8453 UV–VIS spectrophotometer (Waldbronn, Germany).

The hemolysis ratio (hemolysis %) was determined as opposed to a completely hemolyzed solution, employing the following formula [71]:

Hemolysis (%) = (Tested substance absorbance − Negative control absorbance) × 100/(Positive control absorbance − Negative control absorbance)

### 3.7. Evaluation of the In Vivo Biocompatibility of Vesicles Entrapping APAP

To assess the biocompatibility of substances in the nano- or micro-range, in vivo testing involved evaluating the biochemical and hematological profiles of animals exposed to the test substances, including double-distilled water, APAP, and APAP-v-.

Before the experiment, the mice were placed on an elevated wire mesh within a transparent plastic box and allowed to acclimate to the test chamber for a period of 2 h. The testing was conducted between 8 and 12 a.m.

The mice were acclimatized to the laboratory environment for 3 days and were used for only one procedure to ensure consistent conditions. Random assignment was performed, resulting in four groups of six animals each. The animals were administered a single oral dose of substances following the specified treatment regimen:

Group 1 (control): double-distilled water 0.1 mL/10 g body weight;

Group 2 (CS): 0.1 mL/10 body weight CS;

Group 3 (APAP): 150 mg/kg body weight APAP;

Group 4 (APAP-v): CS-based vesicles incorporating APAP, 150 mg/kg body weight;

In the experiment, hematological and biochemical assessments were conducted at two specific time points: 24 h and 7 days following the administration of the substances. Blood samples (0.3 mL) were collected from one of the lateral caudal veins of the mice for analysis. This collection procedure, performed under general anesthesia using 5% isoflurane, is swift (lasting 10–15 s) and does not cause stress to the animals [71,72].

The evaluation of the biocompatibility of APAP-v focused on investigating their impact on various parameters. These included the assessment of full blood count, serum biochemical markers such as liver enzyme activity (alanine aminotransferase—ALT, aspartate aminotransferase—AST, lactate dehydrogenase—LDH), as well as serum urea and creatinine levels. Additionally, certain immunological parameters were examined, namely the phagocytic capacity of peripheral blood polymorphonuclear neutrophils (PC), serum opsonic capacity (OC), and bactericidal capacity of peritoneal macrophages (BC). For the biochemical assessments, fasting venous blood samples collected on heparin were used. The determination of full blood count, as well as the levels of GTP, GOT, and LDH, was performed using a Hematology Analyzer 5 DIFF model BF-5180 (DIRUI, Istanbul, Turkey). To evaluate the PC and BC capacity of peritoneal macrophages, the animals were sacrificed under anesthesia with 5% isoflurane. Peritoneal macrophages were obtained through peritoneal lavage using HANKS solution maintained at a temperature of 37 °C. Thereafter, the samples were centrifuged at 1000 rpm for 10 min. Subsequently, they were exposed to various cultures of *Staphylococcus aureus 94* in a 0.2% glucose broth solution, diluted 1:1000 with saline solution. The specimens were cultured for a 48 h period at a temperature of 37 °C. Afterwards, they were re-plated onto culture media, and subsequent identification of colony formation on plates was conducted.

The animals were sacrificed and liver and kidney fragments were harvested for histopathological evaluation. These tissue specimens were preserved in a 10% formalin solution, subsequently embedded in paraffin, sectioned at 5 μm intervals, and stained with hematoxylin-eosin (H&E). The microscopic analysis of the slides was conducted using a Nikon TI Eclipse optical microscope (Tokyo, Japan), and the images were captured using a Nikon Coolpix 950 digital camera with a resolution of 1600 × 1200 (1.92 Mpx) and optical zoom of x3.

### 3.8. Data Analysis

The results are reported as the mean values ± standard deviation (S.D.) of the mean. Data analysis and statistical processing were conducted using the SPSS 17.0 software, using the ANOVA one-way method for multivariable data analysis. To assess the significance of differences between groups, Newman–Keuls and Tukey post hoc tests were used for multiple comparisons. Statistical significance was indicated for *p* (probability) values below 0.05 compared to the control group.

## 4. Conclusions

We developed a new approach to create CS-coated lipid vesicles loaded with APAP and thoroughly characterized their physicochemical and structural properties. We successfully obtained unique lipid vesicles coated with CS, capable of efficiently encapsulating APAP, while maintaining moderate stability in colloidal dispersion and displaying a prolonged drug in vitro and in vivo release. Initial assessments, including a blood hemolysis assay, indicated promising in vitro biocompatibility of the APAP-v.

The APAP-v were shown to be biocompatible, showing no significant disturbances in hematological, biochemical and immune parameters, after oral administration in mice. Additionally, the histological assessment revealed no notable changes in the configuration of liver and kidney tissues in mice treated with APAP-v, as compared to control animals. These investigations suggest that APAP-v are promising in vivo drug delivery vehicles, holding great potential for future medical applications.

## Figures and Tables

**Table 1:** pH values of the solutions containing APAP.

Solution	pH
APAP solution (APAP)	6.00
APAP vesicles with CS (prior to the dialysis process) (APAP-v nondialyzed)	4.65
Dialyzed APAP vesicles with CS (APAP-v)	6.63

**Table 2:** In vitro hemocompatibility of CS, APAP, APAP-v. Data are displayed as arithmetic mean ± S.D. of the mean values for six animals in a group. ** *p*  <  0.01 statistically significant when compared to the negative control group.

Group	Triton X-100	Negative Control	CS	APAP	APAP-v
Hemolysis %	88.45 ± 4.17 **	0.07 ± 0.01	1.64 ± 0.15	2.38 ± 0.25	2.52 ± 0.21

**Table 3:** The white blood count in CS, APAP, APAP-v groups. The data are displayed as arithmetic mean ± S.D. of the average values for 6 animals per group.

Groups.	Time Elapsed	White Blood Count				
		%				
		PMN	Ly	E	M	B
Control	24 h	19.57 ± 3.23	78.20 ± 4.59	0.35 ± 0.17	1.67 ± 1.13	0.21 ± 0.03
	7 days	19.96 ± 2.59	77.31 ± 3.72	0.41 ± 0.26	2.11 ± 1.42	0.21 ± 0.07
CS	24 h	18.35 ± 2.57	79.45 ± 4.42	0.45 ± 0.35	1.55 ± 1.59	0.20 ± 0.05
	7 days	19.17 ± 1.71	78.41 ± 1.82	0.53 ± 0.42	1.68 ± 1.67	0.21 ± 0.07
APAP	24 h	18.70 ± 3.03	78.47 ± 2.87	0.55 ± 0.23	2.07 ± 1.03	0.21 ± 0.11
	7 days	19.67 ± 2.01	77.43 ± 2.23	0.47 ± 0.59	2.15 ± 1.26	0.22 ± 0.13
APAP-v	24 h	20.84 ± 3.81	75.96 ± 3.71	0.58 ± 0.87	2.41 ± 0.42	0.21 ± 0.07
	7 days	21.42 ± 4.07	76.10 ± 3.23	0.57 ± 0.76	2.35 ± 0.70	0.20 ± 0.18

## Data Availability

Data are contained within the article.
